# Thermo-Mechanical Characterization of Innovative Side-Rounded Superelastic CuNiTi Orthodontic Wires

**DOI:** 10.3390/jfb17090433

**Published:** 2026-08-31

**Authors:** Michele Basilicata, Giovanni Bruno, Gennaro Salvatore Ponticelli, Patrizio Bollero, Raffaella Docimo, Alessandra Fava, Stefano Guarino, Roberto Montanari, Alessandra Palombi, Alfio Scuderi, Alessandra Varone

**Affiliations:** 1 Department of Experimental Medicine and Surgery, UOSD Special Care Dentistry, University of Rome Tor Vergata, 00133 Rome, Italy; 2 Department of Neuroscience, University of Padua, 35122 Padova, Italy; giovanni.bruno.1@unipd.it; 3 Department of Engineering, University Niccolò Cusano, 00166 Rome, Italy; stefano.guarino@unicusano.it (S.G.); alfio.scuderi@unicusano.it (A.S.); 4 Department of Systems Medicine, UOSD Special Care Dentistry, University of Rome Tor Vergata, 00133 Rome, Italy; patrizio.bollero@ptvonline.it; 5 Department of Surgical Sciences, UOSD Pediatric Dentistry, University of Rome Tor Vergata, 00133 Rome, Italy; raffaella.docimo@ptvonline.it; 6 Department for Sustainability, Research Centre of Casaccia, National Agency for New Technologies, Energy and Sustainable Economic Development, Via Anguillarese 301, Santa Maria di Galeria, 00123 Rome, Italy; alessandra.fava@enea.it; 7 Department of Industrial Engineering, University of Rome Tor Vergata, 00133 Rome, Italy; roberto.montanari@uniroma2.it (R.M.); alessandra.palombi@uniroma2.it (A.P.); alessandra.varone@uniroma2.it (A.V.)

**Keywords:** orthodontic wires, CuNiTi shape memory alloy, superelasticity, three-point bending, differential scanning calorimetry

## Abstract

This study evaluates the thermal and mechanical behavior of innovative copper–nickel–titanium (CuNiTi) rectangular orthodontic wires featuring rounded edges across two different sizes for clinical applications. Differential scanning calorimetry (DSC) was performed from −20 °C to +80 °C to determine phase transformation temperatures according to ASTM F2004. Three-point bending tests were conducted in accordance with ISO 15841 to evaluate unloading forces (at 3, 2, 1, and 0.5 mm deflections) and permanent residual deflection at 23 °C (room temperature) and 37 °C (oral environment). DSC analysis revealed a single-stage phase transformation within the range 10 °C to 27 °C, indicating near-superelastic behavior at room temperature. Mechanical testing demonstrated reproducible force-deflection profiles and low permanent deformation. At body temperature (37 °C), unloading forces in the 2.0–0.5 mm activation range averaged between 1.9 N and 3.2 N depending on wire cross-section. In conclusion, the evaluated CuNiTi wires provide consistent force delivery during unloading at physiological temperature, offering suitable mechanical properties for orthodontic alignment applications.

## 1. Introduction

Orthodontic tooth movement is a biologically regulated process induced by controlled mechanical loading of the periodontal ligament, which triggers a coordinated sequence of bone resorption and apposition [1,2,3]. The magnitude, continuity, and stability of the applied forces play a fundamental role in determining treatment efficiency, patient comfort, and the risk of undesirable side effects, such as root resorption and periodontal tissue damage [4,5]. Experimental and clinical evidence indicates that light and continuous forces promote the most favorable biological response while minimizing adverse effects [4,6].

Nickel–titanium (NiTi) shape memory alloys are widely recognized as the material for the initial stages of orthodontic treatment owing to their unique combination of shape memory effect, superelasticity, and biocompatibility [7,8,9]. These functional properties arise from a reversible thermoelastic martensitic transformation between the high-temperature austenitic (B2) phase and the low-temperature martensitic (B19′) phase, which can be induced by temperature or by the application of mechanical stress. Depending on alloy composition and thermomechanical processing, the transformation may also involve an intermediate R-phase [10,11,12]. During mechanical loading, stress-induced martensitic transformation enables NiTi archwires to undergo large recoverable deformations while maintaining an almost constant unloading force over a wide range of deflections [7,9]. Consequently, NiTi archwires are considered biologically favorable for efficient tooth movement while minimizing discomfort and reducing the risk of periodontal tissue damage and root resorption [4,5,13].

However, conventional binary NiTi alloys still exhibit some limitations, including relatively broad transformation hysteresis, variability in transformation temperatures, and sensitivity to thermomechanical processing. To overcome these limitations, several ternary NiTi alloys have been developed through the addition of alloying elements (Au, Cu, Hf, Pd, Pt, V, and Zr) to tailor the functional properties of NiTi for different engineering and biomedical applications [10,14,15,16]. Among these, copper has proved particularly promising for orthodontic applications because it reduces transformation hysteresis, improves cyclic stability and fatigue resistance [11,16], and decreases the sensitivity of transformation temperatures to small compositional variations [11,14].

Furthermore, the thermo-mechanical response of CuNiTi alloys is strongly influenced by the copper content. Increasing the Cu concentration progressively modifies the martensitic transformation sequence from the conventional B2→B19′ transformation observed in binary NiTi alloys to a two-stage B2→B19→B19′ transformation at intermediate Cu contents, and finally to a direct B2→B19 transformation at higher Cu concentrations [14,17,18,19]. These crystallographic changes result in a narrower transformation temperature interval, improved transformation stability, and flexibility in tailoring the functional response of the alloy for biomedical applications. Consequently, CuNiTi orthodontic archwires generally exhibit a more stable thermo-mechanical behavior and more reproducible unloading force plateaus than conventional NiTi wires [8,11,13]. Moreover, Cu enables the austenite finish temperature close to the intraoral temperature, resulting in more predictable force delivery under clinical conditions [8,13].

In addition to alloy composition, the geometry of the archwire represents another key parameter governing orthodontic performance [20]. Cross-sectional dimensions directly influence flexural rigidity, load–deflection behavior, torque expression, and force delivery, whereas edge geometry may affect bracket engagement and friction during sliding mechanics [20,21]. Since flexural rigidity increases with cross-sectional dimensions, wire size represents one of the principal parameters available to clinicians for tailoring orthodontic force delivery according to the stage of treatment [20]. More recently, rectangular CuNiTi archwires featuring rounded edges have been introduced to combine the torque control of conventional rectangular wires with improved bracket engagement and reduced friction during sliding mechanics. However, despite their increasing clinical use, limited information is available regarding the influence of both cross-sectional dimensions and rounded-edge geometry on their thermo-mechanical behavior and clinically relevant force delivery.

Since the clinical performance of thermoactive orthodontic wires is strongly influenced by both phase transformation behavior and wire geometry, comprehensive thermo-mechanical characterization is essential. Differential scanning calorimetry (DSC), standardized according to ASTM F2004 [22], represents the reference technique for determining the characteristic transformation temperatures [12]. Three-point bending tests, performed according to ISO 15841 [23], are widely used to evaluate the force-deflection behavior of orthodontic archwires and to compare their mechanical performance under standardized conditions [24]. The combined use of DSC and three-point bending testing therefore provides a comprehensive understanding of the relationship between phase transformation behavior and clinically relevant mechanical performance.

The aim of the present study was therefore to investigate the thermo-mechanical behavior of a new generation of commercial rectangular CuNiTi orthodontic archwires characterized by an innovative rounded-edge geometry. Differential scanning calorimetry, performed according to ASTM F2004, was employed to determine the characteristic phase transformation temperatures, while standardized three-point bending tests, following ISO 15841, were carried out at room temperature (23 °C) and intraoral temperature (37 °C).

## 2. Materials and Methods

**Figure 1:**
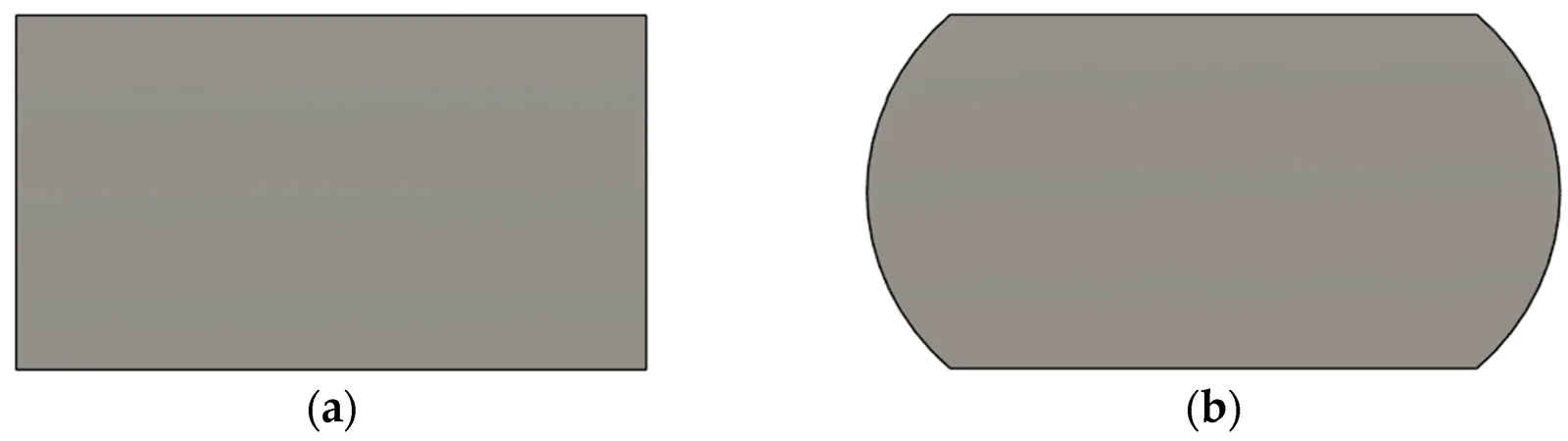
Cross-sectional geometries of: (**a**) conventional rectangular archwires (D1 and D2), and (**b**) novel rounded-edge archwires (DU1 and DU2).

Four commercial orthodontic archwires supplied by Ormco Corporation (Ormco BV Popeweg 72, Venlo, Limburg, The Netherlands) were investigated in as-received condition. Their dimensions are reported in Table 1, while their cross-sectional geometries are shown in Figure 1. The slightly larger major-axis dimension of the rounded-edge archwires is a direct consequence of their cross-sectional geometry.

The chemical composition of the archwires was investigated by field-emission scanning electron microscopy (FEG-SEM, Leo 1530, Zeiss, Oberkochen, Germany) equipped with an energy-dispersive X-ray spectroscopy (EDS) detector (Oxford Instruments, Abingdon, UK).

**Figure 2:**
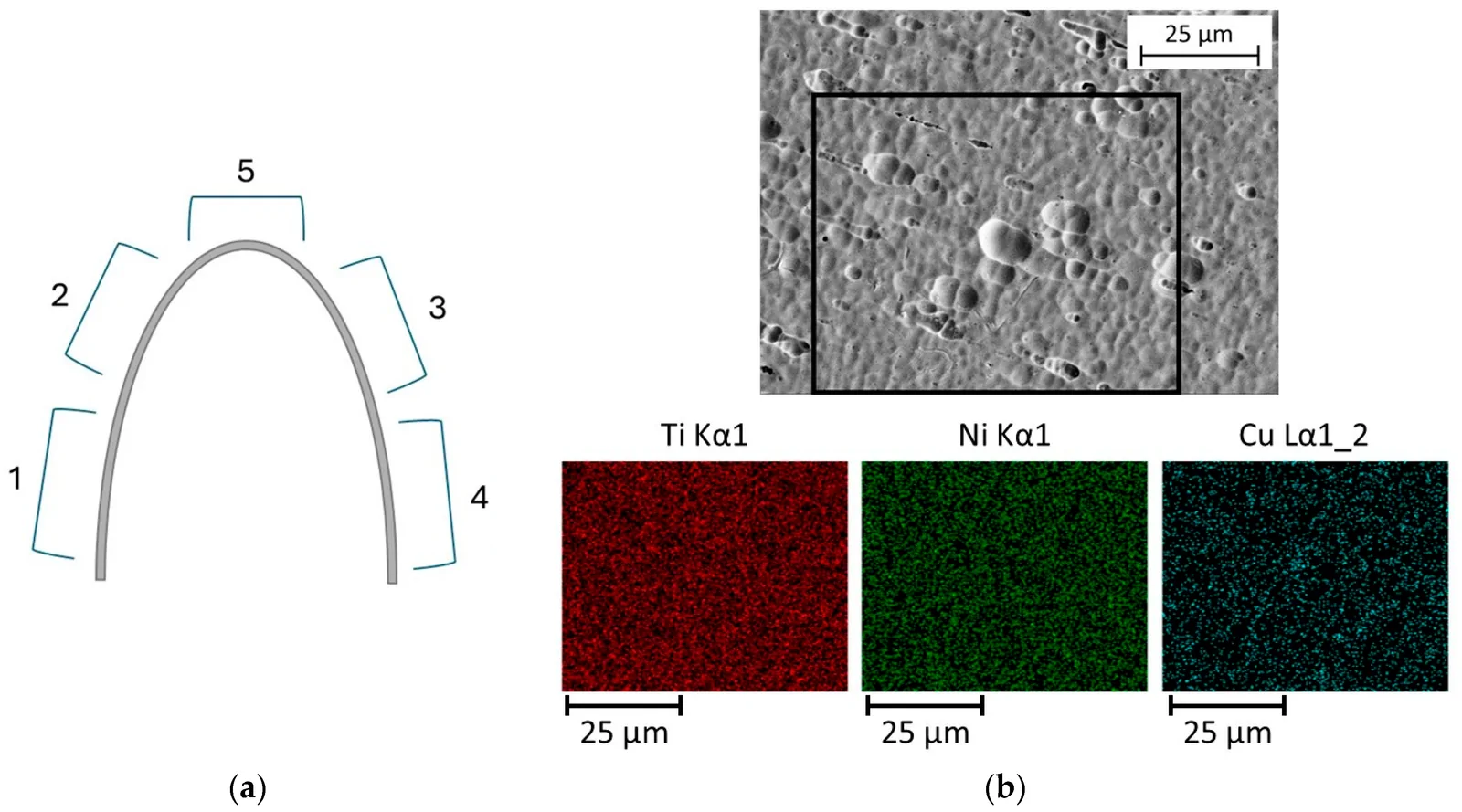
(**a**) Schematic representation of the five regions selected for EDS analysis along orthodontic archwire. (**b**) Representative SEM image with the corresponding EDS maps showing the distribution of Ni, Ti, and Cu.

For each archwire type, EDS characterization was performed on the as-received external surface of one representative archwire in five regions according to the scheme shown in Figure 2a. Ten EDS measurements were acquired within each region to assess possible variations in elemental composition as a function of the position along the archwire. The average chemical composition and corresponding standard deviation reported in Table 2 were calculated from all the individual EDS measurements acquired across the five regions, since no systematic dependence of the Ni, Ti, and Cu contents on the measurement position was observed. As an example, an EDS map of one representative area is reported in Figure 2b. The elemental maps show a homogeneous spatial distribution of Ni, Ti, and Cu, with no evidence of localized elemental segregation.

### 2.1. Thermal Characterization

Differential Scanning Calorimetry (DSC) was performed to determine the phase transition temperatures of the archwires using a Q2000 DSC (TA Instruments, New Castle, DE, USA). Specimen segments weighing approximately 25–40 mg were sealed in standard aluminum pans, according to ASTM F2004. An empty aluminum pan was used as a reference. The thermal cycle was conducted under a constant nitrogen purge gas flow of approximately 20 L/min to guarantee a constant speed of 10 °C/min, both during heating and cooling. The temperature profile was programmed with an initial cooling from room temperature to −20 °C, followed by heating up to +80 °C, and a final cooling step back to −20 °C. To evaluate thermal stability, a single sample for each wire type was subjected to five consecutive thermal cycles. The characteristic transformation temperatures, namely austenite start (*As*), austenite finish (*Af*), austenite peak (*Ap*), martensite start (*Ms*), martensite finish (*Mf*), and martensite peak (*Mp*), were determined from the resulting calorimetric curves using the software TA Universal Analysis 2000 (Version 4.5A).

### 2.2. Mechanical Characterization

**Figure 3:**
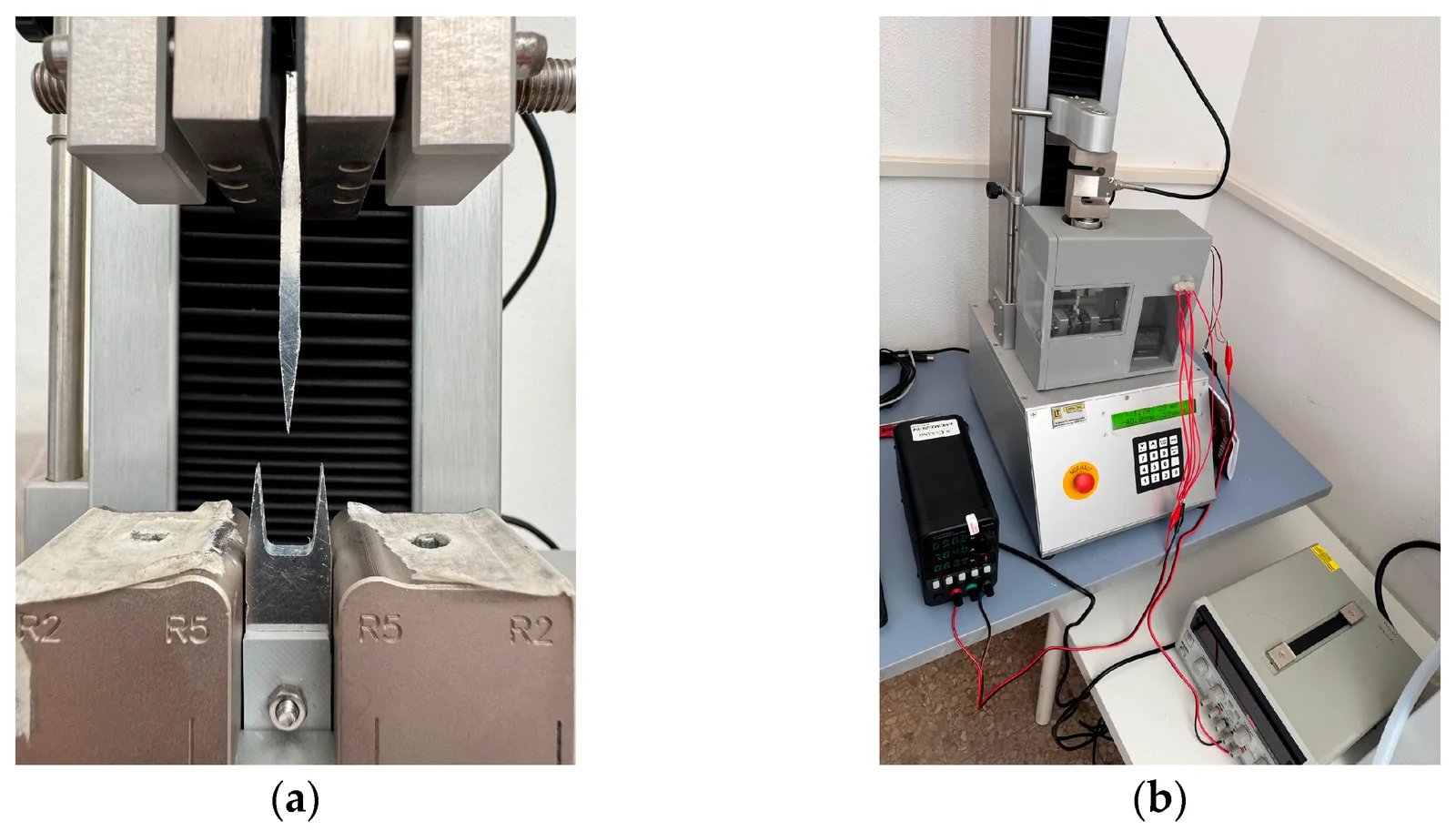
Three-point bending setup: (**a**) piston and supports; (**b**) temperature-controlled chamber.

The mechanical behavior of the archwires was evaluated via a three-point bending test according to ISO 15841 using a Lonos Test universal testing machine (Lonos Test S.r.l, Brescia, Italy) equipped with a 1 kN load cell. For the three-point bending configuration (Figure 3a), the specimens were cut from the straight posterior ends of the archwires to ensure geometric uniformity and eliminate any confounding effects arising from the pre-existing arch curvature. The specimens were then positioned horizontally on two parallel supports with a span distance of 10 mm. The loading piston applied a vertical deflection at the midpoint of the span at a crosshead speed of 1.25 mm/min until a maximum deflection of 3.1 mm was achieved, followed by a controlled unloading cycle back to the baseline. Both piston and supports are characterized by a 0.1 mm radius. The mechanical tests were conducted both at room temperature (23 ± 1 °C) and in a temperature-controlled chamber (Figure 3b) at human intraoral temperature (37 ± 1 °C). The parameters extracted from the load-deflection curves included the unloading plateau force measured at a deflection of 3, 2, 1, and 0.5 mm, and permanent deformation. A sample size of 6 specimens was tested for each condition, yielding a total of 48 archwire segments. All specimens were cut from the straight posterior ends of the archwires for a length of at least 30 mm to ensure geometric uniformity and eliminate any confounding effects from pre-existing arch curvature.

### 2.3. Statistical Analysis

Statistical analysis was performed using the software MATLAB (Version R2026a Update 3). It was based on Analysis of Variance (ANOVA) and carried out to evaluate the influence of the selected process parameters (i.e., Temperature and Wire type) on the investigated response variables (i.e., deflection load and permanent deflection), following the approach proposed by Coleman and Montgomery [25]. The factor ‘Wire’ was analyzed as a four-level categorical variable representing the four distinct commercial archwire configurations (D1, DU1, D2, DU2). This approach evaluates the overall mechanical performance of each as-received commercial product class, reflecting the combined effect of its nominal cross-sectional dimensions and edge geometry. Prior to the analysis, the validity of the ANOVA assumptions was verified through residual analysis, assessing the independence of observations, the normality of residuals, and the homogeneity of variances across the different factor levels. The ANOVA results are reported in terms of degrees of freedom (DoF), adjusted sums of squares (Adj.SS), adjusted mean squares (Adj.MS), F-statistics, *p*-values, and percentage contribution (Π%). The F-statistic was obtained by dividing the adjusted mean square of each factor by the adjusted mean square of the residual error, providing a measure of the relative effect of each process parameter on the response. Higher F-values, exceeding the corresponding critical values (i.e., 4.047 and 2.802 for 1 DoF and 3 DoF, respectively), indicate a statistically stronger influence of the factor under consideration. Statistical significance was assessed using a confidence level of 95% (α = 0.05), with factors considered significant when the corresponding *p*-value was below 0.05. The percentage contribution (Π%) was calculated as the ratio between the adjusted sum of squares of each factor and the total adjusted sum of squares, thereby quantifying the proportion of the overall response variability explained by each parameter. To facilitate the interpretation of the statistical results, main effects and interaction plots are also provided, illustrating the average response values as a function of the different factor levels.

## 3. Results

**Figure 4:**
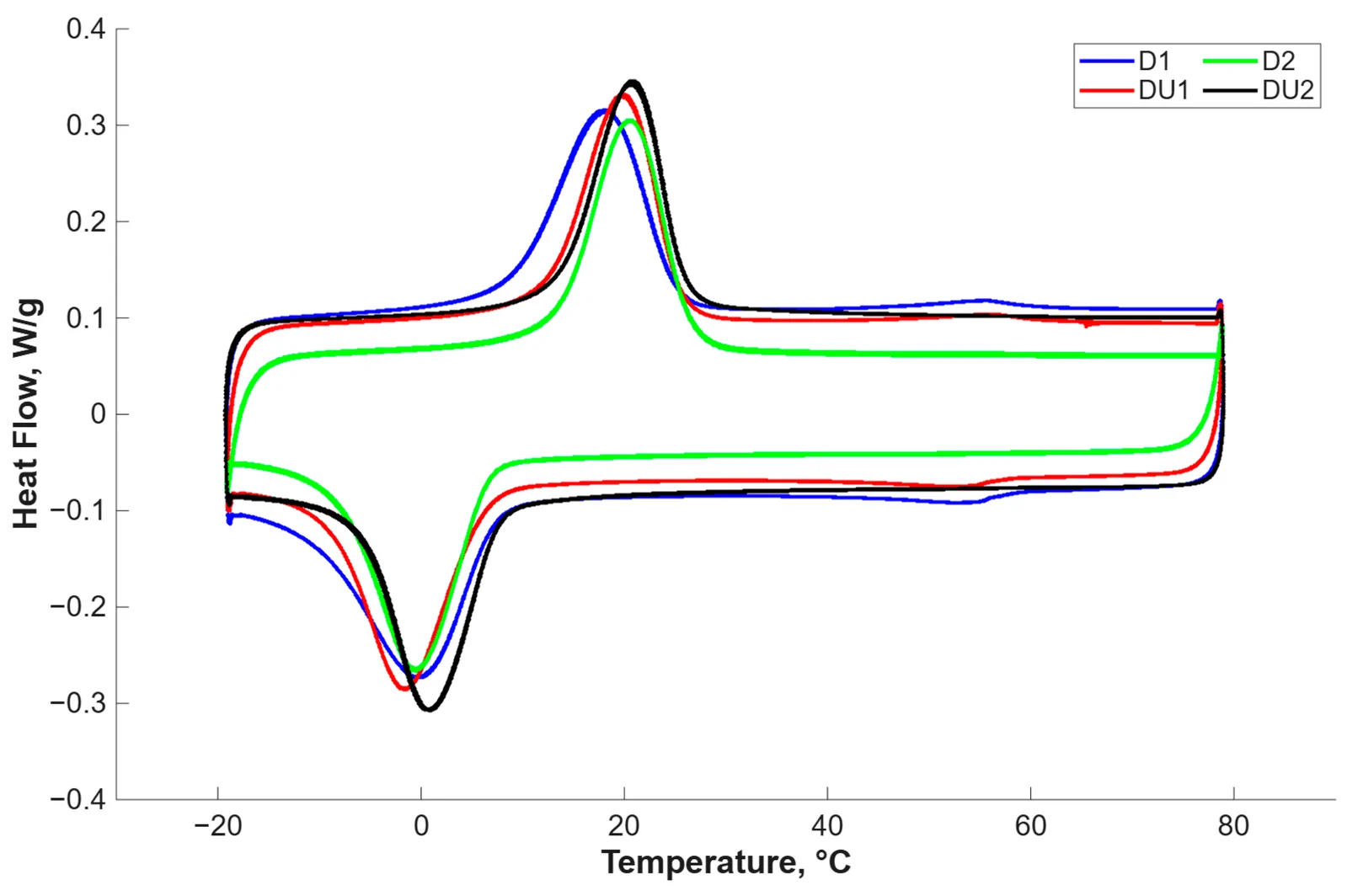
DSC diagram for the four archwires.

The thermo-mechanical characterization of the four CuNiTi archwires (Table 1) is presented below. Differential scanning calorimetry (Figure 4) was first performed to determine the phase transformation temperatures of each wire, providing the thermal basis for interpreting the subsequent mechanical behavior.

All archwires exhibited a single-stage transformation with very similar transformation temperatures (Table 3). The Af temperature ranged between 25.06 and 26.31 °C, indicating that all wires are expected to be fully austenitic under intraoral conditions.

**Figure 5:**
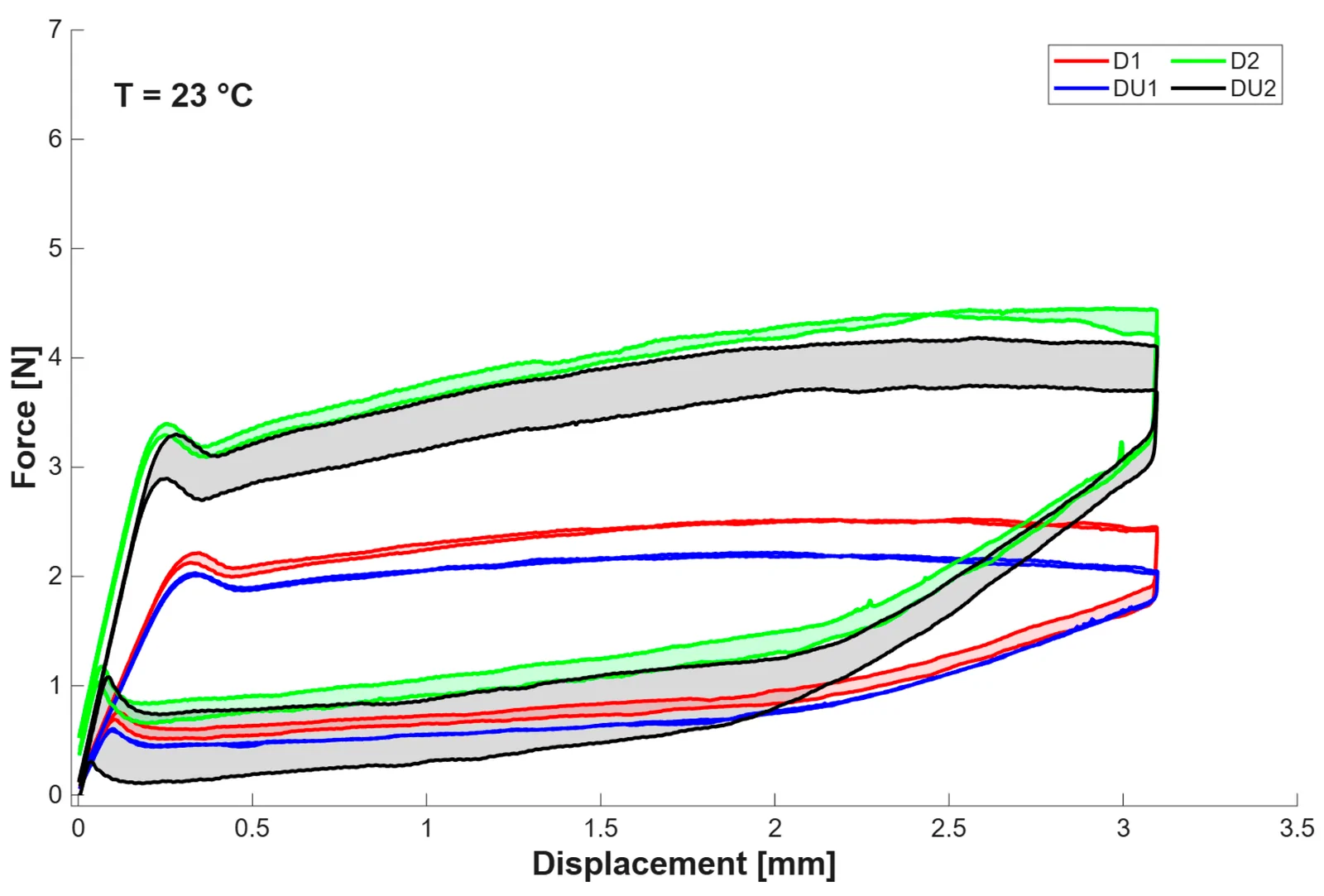
Force–displacement curves for wires tested at room temperature.

**Figure 6:**
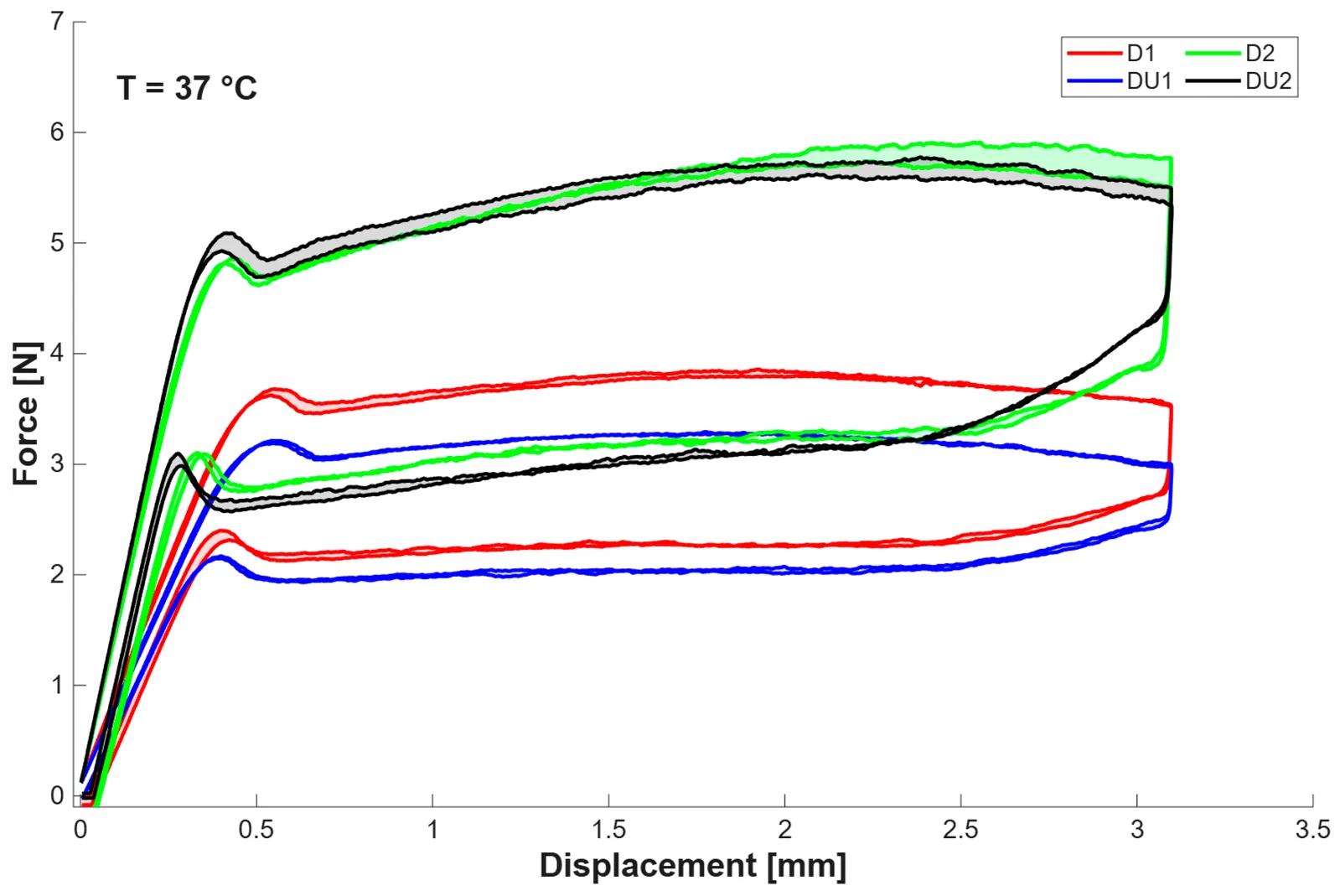
Force–displacement curves for wires tested at intraoral temperature.

Three-point bending tests were then conducted at both room temperature (23 °C) (Figure 5) and intraoral temperature (37 °C) (Figure 6) to evaluate load-deflection behavior, unloading forces at four clinically relevant deflection levels (3, 2, 1, and 0.5 mm), and permanent residual deflection (Table 4).

**Figure 7:**
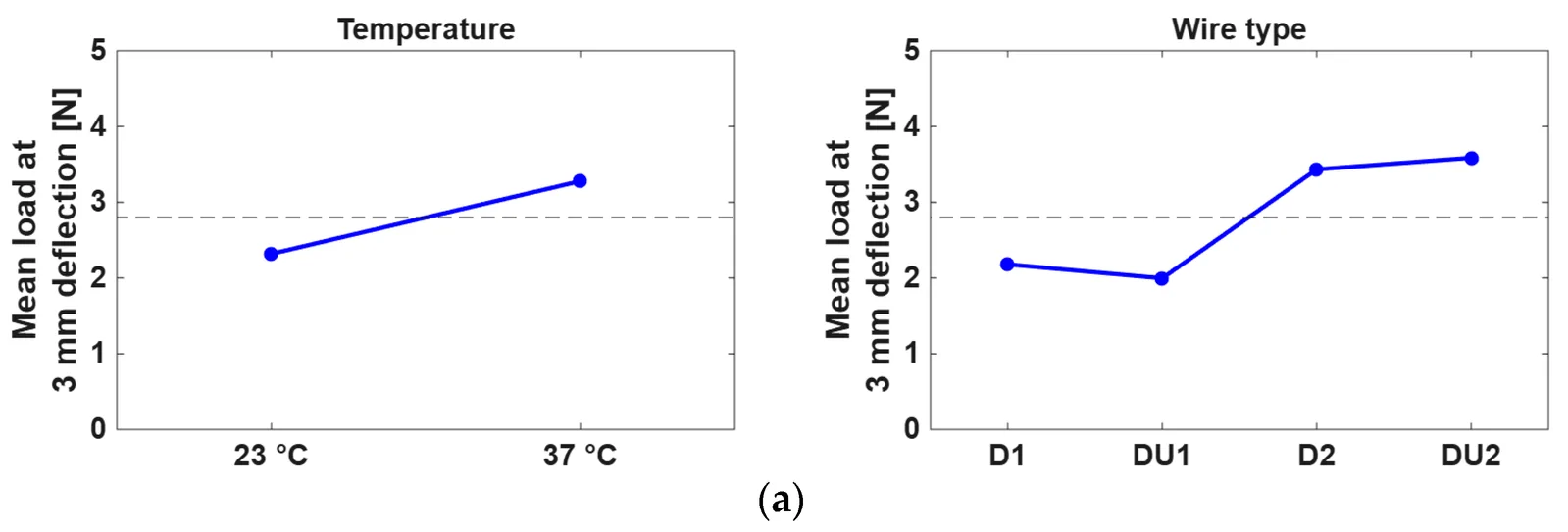

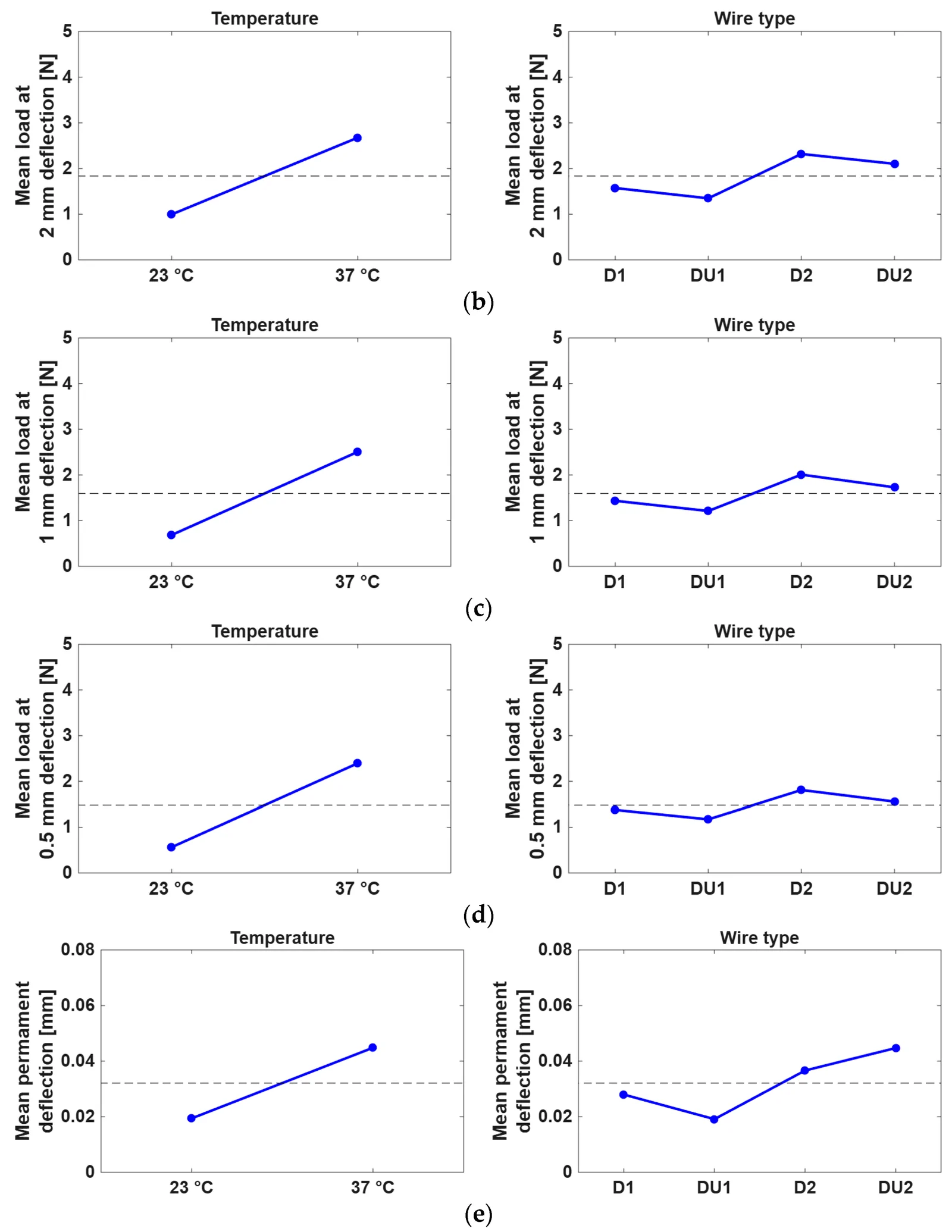
Main effect plots: (**a**) 3 mm deflection; (**b**) 2 mm deflection; (**c**) 1 mm deflection; (**d**) 0.5 mm deflection; (**e**) permanent deflection. The dashed line represents the overall mean.

**Figure 8:**
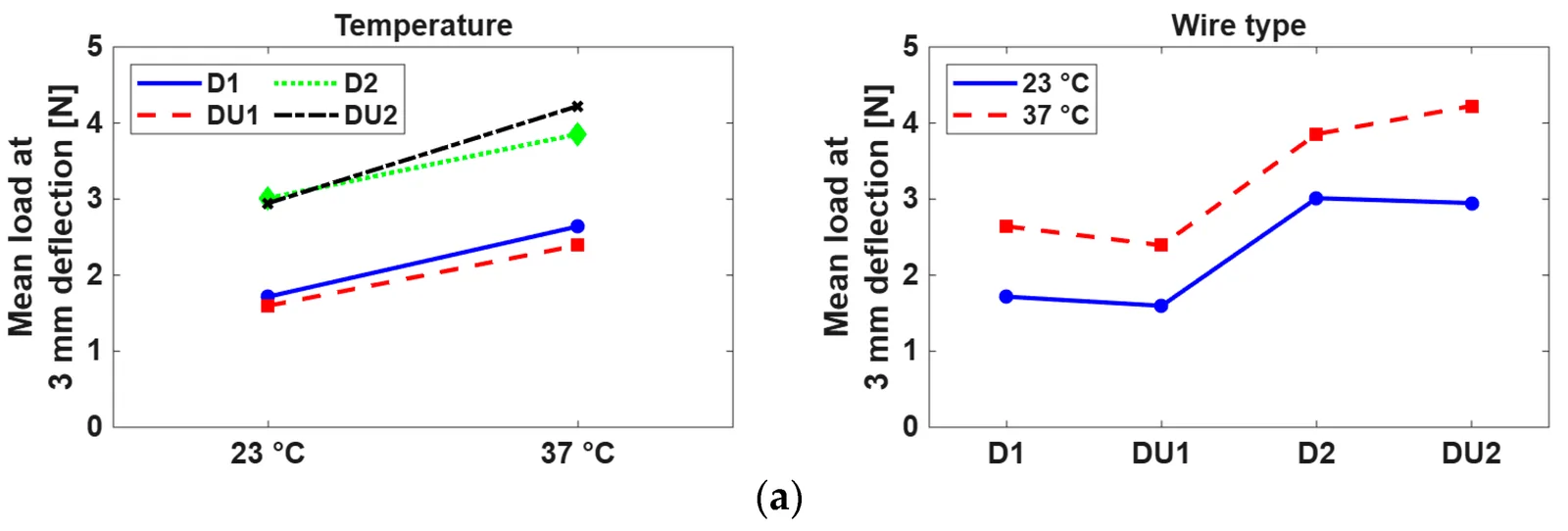

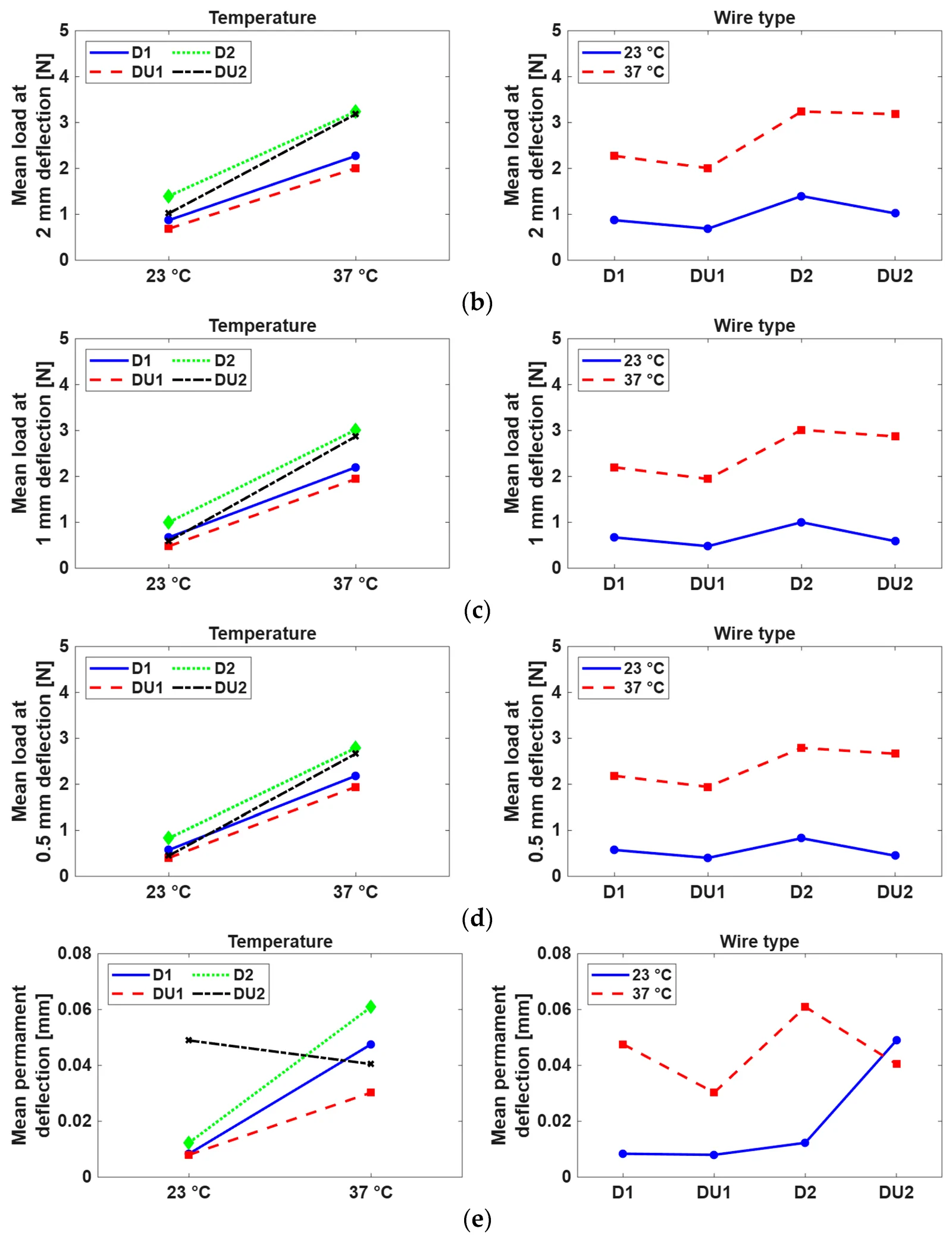
Interaction plots: (**a**) 3 mm deflection; (**b**) 2 mm deflection; (**c**) 1 mm deflection; (**d**) 0.5 mm deflection; (**e**) permanent deflection.

Two-way analysis of variance (ANOVA), considering temperature and wire type as fixed factors, was performed at each deflection level to assess their relative contribution and potential interaction effects on unloading force and permanent deformation. Table 5 shows the ANOVA results, including only the statistically significant terms, i.e., F-value greater than the critical F-value, *p*-value < 0.05, and contribution percentage > 5%. The complete tables are provided in the Appendix A. Figure 7 and Figure 8 show the main effects plots and the interaction plots, respectively.

## 4. Discussion

The present findings confirm that CuNiTi archwires exhibit transformation temperatures close to physiological conditions and deliver stable, low-magnitude unloading forces within the clinically relevant activation range.

EDS analyses revealed comparable average surface compositions for all investigated archwires, with Cu contents ranging from 7.1 to 7.8 wt.%. These results are consistent with the DSC data, which showed very similar transformation temperatures for all archwires, and a single-stage transformation occurring between approximately 10 °C and 26 °C, indicating that the alloy is fully austenitic at oral temperature. This thermal profile is consistent with data commonly reported for commercial Cu-NiTi alloys, in which copper narrows and stabilizes the transformation interval compared with conventional superelastic NiTi [14,16,17]. The thermal hysteresis, calculated as the difference between the transformation peak temperatures, was approximately 20 °C for all investigated archwires, in agreement with values commonly reported for commercial CuNiTi orthodontic alloys [26,27,28]. In addition, the excellent thermal stability of CuNiTi alloys, with almost perfect superimposition of the curves at each heating/cooling cycle (Figure 4), represents a significant advantage, indicating excellent reproducibility of the thermoelastic transformation over repeated heating/cooling cycles.

The mechanical data corroborate and extend this thermal behavior. At every deflection level, unloading forces at 37 °C were consistently and substantially higher than at 23 °C. This is best explained by the temperature dependence of the transformation stress typical of thermoelastic shape-memory alloys. In fact, once the material is heated above its transformation interval, the stress required to induce the martensitic transformation increases, raising the unloading plateau accordingly. Because the DSC-derived transformation range (i.e., 9–27 °C) lies below oral temperature, the wires operate in a fully austenitic, higher-plateau regime intraorally, whereas at room temperature the incomplete transformation yields lower, near-superelastic force output. This thermo-mechanical coupling is in line with previous reports correlating DSC transformation temperatures with clinically measured unloading forces [11,14].

At room temperature, the rounded-edge wires delivered systematically lower unloading forces than their conventional counterparts, e.g., at 2 mm, DU1 was ~21% lower than D1, and DU2 was ~27% lower than D2. This trend was consistent across all four deflection levels at 23 °C. At 37 °C, however, the difference between paired wires narrowed considerably, e.g., DU1 was ~12% lower than D1, and DU2 was ~2% lower than D2. Moreover, at 3 mm deflection the trend even reversed for the thicker pair, with DU2 exceeding D2. This pattern shows that rounded-edge geometry affects flexural stiffness in a temperature-dependent manner. The effect of cross-sectional shape is most pronounced at room temperature. Moreover, it is worth noting that at room temperature, the DU2 group displayed a mean permanent deflection of 0.049 ± 0.085 mm. This stems from the fact that two specimens achieved almost complete strain recovery, while the remaining four specimens exhibited varying levels of permanent deformation. The presence of these quasi-zero-value recoveries alongside non-zero values contributes directly to the high standard deviation relative to the mean. This phenomenon can be attributed to the temperature dependency of the critical stress required for martensitic transformation. At 23 °C, ambient thermal energy is insufficient to ensure full shape memory recovery upon load release. Under mechanical loading, stress-induced martensite forms, however, during unloading, a fraction of this structure remains stabilized in the lattice due to insufficient thermodynamic driving force to trigger complete reverse transformation to austenite. Furthermore, the altered cross-sectional geometry of DU2, featuring rounded corners, modifies the internal stress gradient across the wire during bending. At oral temperature, this geometric effect is largely masked because the wire becomes fully austenitic, and the stress generated by the phase transformation dominates the overall force response.

The ANOVA results provide further mechanistic insight into how temperature and wire geometry jointly determine force delivery. At larger deflections, wire cross-sectional dimension was the dominant source of variance (67.5%). Since this factor spans both the two size classes and the edge-geometry pairing within each class, this large contribution primarily reflects the geometry-driven stiffness difference between the two thickness classes, consistent with the pairwise comparisons above showing a secondary, temperature-dependent effect of edge rounding within each class. However, as deflection decreased toward the clinically relevant range, the relative contribution of temperature increased markedly, while the influence of wire dimension declined proportionally. This pattern suggests that within the activation interval most relevant to initial alignment (2–0.5 mm), the phase composition of the alloy, and thus intraoral temperature, becomes the principal determinant of the force delivered to the tooth, with wire size and edge geometry playing a comparatively minor role. Notably, while the interaction between temperature and wire type was statistically significant across all deflection ranges, its percentage contribution to total variance remained small (1.17% to 3.25%). This suggests that although an interaction exists, thermal transformation and geometric factors operate largely independently from a practical perspective.

From a biological standpoint, the unloading forces recorded within the clinical range 2.0–0.5 mm at 37 °C is approximately 1.9–3.2 N depending on wire dimension. In relation to the commonly cited biologically favorable window of 0.2–1.5 N [4,5], while the thinner 0.014-inch wires approached this range more closely, the 0.018-inch wires delivered forces toward the upper boundary or beyond it. Within each thickness class, the rounded-edge archwire (DU1, DU2) tended to deliver slightly lower, and thus biologically more favorable, forces than its conventional counterpart at room temperature, though this advantage diminished at intraoral temperature. These findings, although not directly validated in clinical settings, suggest that clinicians may consider both wire size and, to a lesser extent, edge geometry when selecting wires for patients who may be particularly sensitive to root resorption or discomfort. The observed mechanical profiles could potentially contribute to reducing adverse tissue responses; however, further clinical studies are needed to determine whether these biomechanical characteristics translate into clinically relevant benefits. Nevertheless, all wires exhibited an extremely low permanent residual deflection, i.e., lower than 0.061 mm even at 37 °C, confirming excellent shape recovery and superelastic stability across the tested temperature range. Notably, the ANOVA for permanent deflection showed that temperature, although statistically significant, accounted for a comparatively small proportion of variance, while wire dimension and its interaction with temperature were not significant, and most of the variability was attributable to residual error, likely reflecting the comparatively high dispersion observed for DU2 at 23 °C, which should be interpreted with caution given its wide standard deviation. This indicates that, unlike unloading force, permanent deformation is largely independent of both wire thickness and edge geometry and only marginally affected by temperature, reinforcing the robustness of the pseudoelastic recovery mechanism across clinically relevant conditions.

These findings should be interpreted considering the limitations inherent to single-cycle three-point bending tests, which, despite being standardized [23], do not fully replicate the complex intraoral environment where friction, bracket geometry, interbracket distance, and cyclic loading modulate wire behavior over time [29]. Additionally, as previously reported for other CuNiTi archwires, batch-to-batch variability in manufacturing may influence transformation temperatures and force output, and the present results, obtained on a single production batch, should be confirmed through inter-batch and cyclic-loading investigations. Furthermore, a fundamental limitation is the direct comparison between conventional rectangular and side-rounded wires of the same nominal class. However, in alignment with ISO 15841 testing protocols, raw flexural forces were evaluated rather than normalized stresses, as absolute force reflects the actual mechanical output experienced in clinical practice. Future studies incorporating simulated intraoral friction, long-term cyclic testing, and clinical validation would help translate these thermo-mechanical findings into more definitive guidance for archwire selection during the initial alignment phase. Moreover, tensile strength testing will be carried out to evaluate the alloy’s potential under broader mechanical standards [30].

## 5. Conclusions

The present study provides a comprehensive thermo-mechanical characterization of innovative rounded-edge CuNiTi orthodontic archwires, demonstrating that their cross-sectional modification directly influences their force delivery profile and phase transformation response. The combination of transformation temperatures below intraoral conditions, stable pseudoelastic behavior, and excellent elastic recovery enables predictable unloading force delivery while maintaining negligible permanent deformation.

By integrating differential scanning calorimetry, standardized three-point bending tests, and statistical modelling, this study provides new insights into the mechanisms governing force delivery in side-rounded CuNiTi archwires. In particular, the findings demonstrate that, within the clinically relevant activation range, temperature exerts a greater influence on unloading forces than wire dimension, highlighting the dominant role of phase transformation in determining the biomechanical response under intraoral conditions.

Within the limitations of an in vitro setup, these mechanical findings suggest that side-rounded CuNiTi wires represent a distinct option for force delivery in orthodontic applications. The availability of paired wire configurations, matched in thickness but differing in edge geometry, provides a mechanical framework that could theoretically offer alternative force levels within a given thickness class while preserving the pseudoelastic characteristics of the alloy. However, whether these benchtop mechanical differences translate into clinical advantages remains to be confirmed. Further investigations under simulated intraoral conditions, together with prospective clinical studies, are warranted to validate the long-term clinical performance and therapeutic benefits of these innovative archwires.

## Figures and Tables

**Table 1:** CuNiTi orthodontic archwires dimensions.

Archwire	Abbreviation	Size [in]	Size [mm]
Damon™ 0.014 × 0.025	D1	0.014 × 0.025	0.356 × 0.635
Damon™ Ultima 0.014 × 0.0275	DU1	0.014 × 0.0275	0.356 × 0.699
Damon™ 0.018 × 0.025	D2	0.018 × 0.025	0.457 × 0.635
Damon™ Ultima 0.018 × 0.0275	DU2	0.018 × 0.0275	0.457 × 0.699

**Table 2:** Average chemical composition of CuNiTi archwires (wt%). Values are reported as the mean ± standard deviation calculated from the EDS measurements acquired across the entire archwire.

Archwire	Ni	Ti	Cu
D1	50.3 ± 0.6	42.4 ± 0.3	7.3 ± 0.5
DU1	49.9 ± 0.8	42.3 ± 0.4	7.8 ± 0.5
D2	50.1 ± 0.9	42.3 ± 0.7	7.6 ± 0.7
DU2	50.3 ± 0.5	42.6 ± 0.4	7.1 ± 0.7

**Table 3:** Characteristic transformation temperatures.

Phase	Temperature [°C]			
	D1	DU1	D2	DU2
Austenite start (*As*)	9.19	12.85	13.79	13.70
Austenite finish (*Af*)	25.06	25.47	26.31	25.94
Austenite peak (*Ap*)	18.13	19.84	20.49	20.85
Martensite start (*Ms*)	7.45	6.48	6.71	7.68
Martensite finish (*Mf*)	−9.73	−8.79	−7.98	−5.59
Martensite peak (*Mp*)	−0.19	−1.71	−0.51	0.76

**Table 4:** Mechanical properties. The values are listed as the mean ± standard deviation.

Temperature [°C]	Wire	Deflection Load [N]				Permanent Deflection [mm]
		3 mm	2 mm	1 mm	0.5 mm	
23	D1	1.717 ± 0.050	0.872 ± 0.048	0.671 ± 0.040	0.574 ± 0.040	0.008 ± 0.001
	DU1	1.598 ± 0.075	0.688 ± 0.087	0.479 ± 0.096	0.402 ± 0.088	0.008 ± 0.001
	D2	3.013 ± 0.088	1.398 ± 0.099	1.001 ± 0.093	0.829 ± 0.086	0.012 ± 0.004
	DU2	2.948 ± 0.138	1.021 ± 0.203	0.589 ± 0.255	0.449 ± 0263	0.049 ± 0.085
37	D1	2.641 ± 0.048	2.273 ± 0.065	2.198 ± 0.058	2.183 ± 0.069	0.048 ± 0.006
	DU1	2.393 ± 0.040	2.006 ± 0.051	1.943 ± 0.046	1.942 ± 0.050	0.030 ± 0.006
	D2	3.855 ± 0.157	3.239 ± 0.099	3.014 ± 0.054	2.791 ± 0.052	0.061 ± 0.005
	DU2	4.225 ± 0.034	3.183 ± 0.059	2.870 ± 0.044	2.665 ± 0.043	0.040 ± 0.005

**Table 5:** ANOVA results including only the statistically significant terms, i.e., F-value greater than the critical F-value, *p*-value < 0.05, and contribution percentage > 5%.

Output		Source	F-Value	*p*-Value	Π%
Deflection load	3 mm	Temperature	1140	<0.001	30.28
		Wire	845	<0.001	67.48
	2 mm	Temperature	2800	<0.001	78.59
		Wire	202	<0.001	17.04
	1 mm	Temperature	2810	<0.001	86.35
		Wire	102	<0.001	9.41
	0.5 mm	Temperature	2750	<0.001	90.71
		Wire	60	<0.001	5.95
Permanent deflection		Temperature	6.99	0.012	12.47
		Error	-	-	71.39

## Data Availability

The raw data supporting the conclusions of this article will be made available by the authors on request.

## Abbreviations

The following abbreviations are used in this manuscript:

Adj.MS	Adjusted Mean Sum of Squares
Adj.SS	Adjusted Sum of Squares
Af	Austenite finish
ANOVA	Analysis of Variance
Ap	Austenite peak
As	Austenite start
ASTM	American Society for Testing and Materials
CuNiTi	Copper–Nickel–Titanium
D1	Conventional archwire 0.014 × 0.025 in
D2	Conventional archwire 0.018 × 0.025 in
DoF	Degree of Freedom
DSC	Differential Scanning Calorimetry
DU1	Rounded-edge archwire 0.014 × 0.0275 in
DU2	Rounded-edge archwire 0.018 × 0.0275 in
EDS	Energy-Dispersive X-ray Spectroscopy
F-value	Fisher value
FEG-SEM	Field Emission Gun–Scanning Electron Microscope
ISO	International Organization for Standardization
Mf	Martensite finish
Mp	Martensite peak
Ms	Martensite start
Π%	Contribution percentage

## Appendix A

This section contains the complete ANOVA tables mentioned in Section 3. Please, note that the statistically significant terms, i.e., F-value > critical F-value, *p*-value < 0.05, and contribution percentage > 5% are highlighted in Italic.

**Table A1:** ANOVA table for 3 mm deflection.

Source	DoF	Adj SS	Adj MS	F-Value	*p*-Value	Π%
*Temperature*	*1*	*11.059*	*11.059*	*1140*	*<0.001*	*30.28*
*Wire*	*3*	*24.641*	*8.214*	*845*	*<0.001*	*67.48*
Temperature × Wire	3	0.429	0.143	15	<0.001	1.17
Error	40	0.389	0.010	-	-	1.07
Total	47	36.518	-	-	-	-

**Table A2:** ANOVA table for 2 mm deflection.

Source	DoF	Adj SS	Adj MS	F-Value	*p*-Value	Π%
*Temperature*	*1*	*33.900*	*33.900*	*2800*	*<0.001*	*78.59*
*Wire*	*3*	*7.349*	*2.450*	*202*	*<0.001*	*17.04*
Temperature × Wire	3	1.402	0.467	39	<0.001	3.25
Error	40	0.485	0.012	-	-	1.12
Total	47	43.135	-	-	-	-

**Table A3:** ANOVA table for 1 mm deflection.

Source	DoF	Adj SS	Adj MS	F-Value	*p*-Value	Π%
*Temperature*	*1*	*39.820*	*39.820*	*2810*	*<0.001*	*86.35*
*Wire*	*3*	*4.339*	*1.446*	*102*	*<0.001*	*9.41*
Temperature × Wire	3	1.387	0.463	33	<0.001	3.01
Error	40	0.568	0.014	-	-	1.23
Total	47	46.114	-	-	-	-

**Table A4:** ANOVA table for 0.5 mm deflection.

Source	DoF	Adj SS	Adj MS	F-Value	*p*-Value	Π%
*Temperature*	*1*	*40.254*	*40.254*	*2750*	*<0.001*	*90.71*
*Wire*	*3*	*2.647*	*0.881*	*60*	*<0.001*	*5.95*
Temperature × Wire	3	0.896	0.299	20	<0.001	2.02
Error	40	0.586	0.015	-	-	1.32
Total	47	44.378	-	-	-	-

**Table A5:** ANOVA table for permanent deflection.

Source	DoF	Adj SS	Adj MS	F-Value	*p*-Value	Π%
*Temperature*	*1*	*0.008*	*0.008*	*6.99*	*0.012*	*12.47*
Wire	3	0.004	0.002	1.32	0.282	7.04
Temperature × Wire	3	0.006	0.002	1.70	0.183	9.10
*Error*	*40*	*0.044*	*0.001*	-	-	*71.39*
Total	47	0.062	-	-	-	-
